# Entanglement Detection via Direct-Sum Majorization Uncertainty Relations

**DOI:** 10.1038/s41598-019-57302-0

**Published:** 2020-01-16

**Authors:** Kun Wang, Nan Wu, Fangmin Song

**Affiliations:** State Key Laboratory for Novel Software Technology, Department of Computer Science and Technology, Nanjing University, Nanjing, 210023 China

**Keywords:** Qubits, Qubits, Quantum information, Quantum information

## Abstract

In this paper we investigate the relationship between direct-sum majorization formulation of uncertainty relations and entanglement, for the case of two observables. Our primary results are entanglement detection methods based on direct-sum majorization uncertainty relations. These detectors provide a set of sufficient conditions for detecting entanglement whose number grows linearly with the dimension of the state being detected.

## Introduction

Uncertainty relations is an essential element of quantum mechanics. They characterize intrinsic limitation on our ability to predict the outcomes of noncommuting observables simultaneously. There are various approaches to quantify these relations. The original formulation by Heisenberg^1^ applied the standard deviation to measure the uncertainty for momentum and position operators. This result was then generalized to arbitrary observables^2^. Later the entropic uncertainty relations emerged^3–9^ and found applications in the security analysis of quantum cryptographic protocols. In this approach, uncertainty is measured by entropy functions like Shannon^10^ and Rényi^11^. We refer to^12^ for a comprehensive review on this topic. However, entropies are by no means the most adequate to use. With this motivation, majorization is used to study uncertainty relations^13^. This line of research is further investigated in^14–16^.

Entanglement is one of the most counterintuitive phenomena of quantum mechanics and has been extensively investigated in the past decades^17–19^. Entangled states are key ingredient in quantum information processing, such as quantum teleportation^20^ and dense coding^21^. It is therefore important to decide whether a given quantum state is entangled or not. However, this problem is known to be computationally intractable^22^. As so, computationally tractable necessary conditions for entanglement detection, which provide a partial solution, have been the subject of active research in recent years^23^.

References ^24,25^ present several methods for detecting entanglement via variance based uncertainty relations. Similar methods have been designed using entropy based uncertainty relations^26,27^. One may wonder whether there exists a relationship between the majorization based uncertainty relation and entanglement. The answer is affirmative. In^28^, the author applies the tensor-product majorization formulation of uncertainty^13^ to the problem of entanglement detection. In this paper we use the direct-sum majorization uncertainty relation, developed in^16^, to design an entanglement detection method. As the direct-sum majorization bound has analytical solution while the tensor-product majorization bound does not, our direct-sum majorization based detection method is more practical than the tensor-product majorization based method.

The rest of this paper is structured as follows. We first briefly review the direct-sum majorization formulation of uncertainty. Then, we present our main result — an entanglement detection method based on the direct-sum majorization uncertainty. Example is given to illustrate how our method works.

## Direct-sum Majorization Uncertainty Relations

This section presents a basic review of the majorization theory and the formulation of direct-sum majorization approach to uncertainty relations.

### Majorization

Let ${{\mathbb{R}}}_{+}=[0,\infty )$ be the set of non-negative real numbers, ${{\mathbb{R}}}_{+}^{d}=\{({p}_{1},\ldots ,{p}_{d}):{p}_{i}\in {{\mathbb{R}}}_{+}\}$ be the set of *d*-dimensional real vectors with non-negative components. We denote by $p\in {{\mathbb{R}}}_{+}^{d}$ a *d*-dimensional vector and by *p*_*i*_ the *i*-th element of *p*. For any vector $p\in {{\mathbb{R}}}_{+}^{d}$, let *p*^↓^ be the vector obtained from *p* by arranging the components of the latter in descending order. Given two vectors $p,q\in {{\mathbb{R}}}_{+}^{d}$, *p* is said to be *majorized* by *q* and written $p\,\prec \,q$ if

1 $$\forall k\in \{1,\,\ldots ,\,d-1\},\,\mathop{\sum }\limits_{i=1}^{k}\,{p}_{i}^{\downarrow }\le \mathop{\sum }\limits_{i=1}^{k}\,{q}_{i}^{\downarrow }\,{\rm{and}}\,\mathop{\sum }\limits_{i=1}^{d}\,{p}_{i}^{\downarrow }=\mathop{\sum }\limits_{i=1}^{d}\,{q}_{i}^{\downarrow }.$$

Intuitively, $p\,\prec \,q$ means that the sum of largest *k* components of *p* is no larger than the sum of *k* largest components of *q*. The majorization order is a partial order, i.e., not every two vectors are comparable under majorization. When studying majorization among two vectors of different dimensions, we append 0(s) to the vector with smaller dimension so that two vectors have the same dimension.

A related concept is the *supremum* of a set of *N* vectors, defined as the vector that majorizes every element of the set and, is majorized by any vector that has the same property. We now briefly describe how to construct the supremum vector, more details can be found in^13,29^. Let ${\mathscr{S}}={\{{p}^{(n)}\in {{\mathbb{R}}}_{+}^{d}\}}_{n=1}^{N}$ a set of *N* vectors. To construct the supremum for ${\mathscr {S}}$, we define a (*d* + 1)-dimensional vector Ω with components Ω_0_ = 0, ∀*k* ∈ {1, …, *d*}

2 $${\Omega }_{k}=\,{\rm{\max }}(\mathop{\sum }\limits_{i=1}^{k}\,{[{p}^{(1)}]}_{i}^{\downarrow },\,\ldots ,\,\mathop{\sum }\limits_{i=1}^{k}\,{[{p}^{(N)}]}_{i}^{\downarrow })$$

The desired supremum ${\omega }^{{\rm{\sup }}}:=({\omega }_{1}^{{\rm{\sup }}},\,\ldots ,\,{\omega }_{d}^{{\rm{\sup }}})$ is then given by

3 $$\forall \,k\in \{1,\,\ldots ,\,d\},\,{\omega }_{k}^{{\rm{\sup }}}={\Omega }_{k}-{\Omega }_{k-1}.$$

The construction given in Eq. (3) guarantees that *ω*^sup^ majorizes every element of the set ${\mathscr {S}}$, but *ω*^sup^ does not necessarily appear in a descending order and may, therefore, fails to be majorized by other vectors with the same property. In such case, we must perform a “flattening” process. This process starts with *ω*^sup^ obtained in Eq. (3), and for every pair of components violating the descending order, say, ${\omega }_{k}^{{\rm{\sup }}} < {\omega }_{k+1}^{{\rm{\sup }}}$, replaces the pair by their mean such that the updated two elements are ${\hat{\omega }}_{k}^{{\rm{\sup }}}={\hat{\omega }}_{k+1}^{{\rm{\sup }}}=({\omega }_{k}^{{\rm{\sup }}}+{\omega }_{k+1}^{{\rm{\sup }}})/2$. This process continues until a descending vector corresponding to the supremum is obtained.

### Direct-sum majorization uncertainty

We now briefly introduce the uncertainty relation characterized by direct-sum majorization relation. We remark that the results summarized here are originally presented in^16^.

Let *H* be a *d*-dimensional Hilbert space. Denote by ${\cal{D}} (H)$ the set of quantum states in *H*. Let ${\mathbb{X}}$ and ${\mathbb{Z}}$ be two nondegenerate and noncommuting observables, and *ρ* be a state on *H*. Assume the spectral decompositions of ${\mathbb{X}}$ and ${\mathbb{Z}}$ are given by

4 $${\mathbb{X}}=\mathop{\sum }\limits_{i=1}^{d}\,{\alpha }_{i}|{x}_{i}\rangle \langle {x}_{i}|,\,{\mathbb{Z}}=\mathop{\sum }\limits_{j=1}^{d}\,{\beta }_{j}|{z}_{j}\rangle \langle {z}_{j}|,$$

where {|*x*_*i*_〉} and {|*z*_*j*_〉} are the eigenstates of ${\mathbb{X}}$ and ${\mathbb{Z}}$, respectively. These two set of eigenstates provide two orthonormal bases of *H*. We then define the probability distribution induced by the measurement of observable ${\mathbb{X}}$ for a system in state *ρ* in the usual manner

5 $$p({\mathbb{X}}|\rho )=({p}_{1},\,\ldots ,\,{p}_{d}),\,{p}_{i}=\langle {x}_{i}|\rho |{x}_{i}\rangle .$$

Similarly, measurement of observable ${\mathbb{Z}}$ for the system in state *ρ* induces a probability distribution given by

6 $$q({\mathbb{Z}}|\rho )=({q}_{1},\,\ldots ,\,{q}_{d}),\,{q}_{j}=\langle {z}_{j}|\rho |{z}_{j}\rangle .$$

We are interested in the uncertainty relation induced by these two observables. In^16^ the direct-sum majorization approach is used to is to characterize the uncertainty about $p({\mathbb{X}}|\rho )$ and $q({\mathbb{Z}}|\rho )$:

7 $$\forall \,\rho \in  {\cal{D}} (H),\,p({\mathbb{X}}|\rho )\oplus q({\mathbb{Z}}|\rho )\,\prec \,{\omega }^{{\mathbb{X}}\oplus {\mathbb{Z}}},$$

where ${\omega }^{{\mathbb{X}}\oplus {\mathbb{Z}}}$ is a 2*d*-dimensional vector independent of *ρ* which can be explicitly calculated from observables ${\mathbb{X}}{\rm{and}}{\mathbb{Z}}$. Intuitively, ${\omega }^{{\mathbb{X}}\oplus {\mathbb{Z}}}$ is the supremum vector of the following set

8 $${\mathscr{S}}:=\{p({\mathbb{X}}|\rho )\oplus q({\mathbb{Z}}|\rho )\,:\rho \in  {\cal{D}} (H)\}.$$

Now we show how to compute ${\omega }^{{\mathbb{X}}\oplus {\mathbb{Z}}}$ analytically. From the definitions of *p* and *q*, we see that only the eigenstates of ${\mathbb{X}}$ and ${\mathbb{Z}}$ matter. We define a *d* × *d* unitary operator *U* whose elements are given by ${U}_{ij}=\langle {x}_{i}|{z}_{j}\rangle$. *U* is known as the overlap matrix^16^ as it characterizes the overlap of the two orthonormal bases. For each $k\in \{1,\ldots ,d\}$, let SUB(*U*, *k*) be the set of submatrices of class *k* of *U* defined as

9 $${\rm{SUB}}(U,k)=\{M\,:M\,{\rm{is}}\,{\rm{a}}\,{\rm{submatrix}}\,{\rm{of}}\,U\,{\rm{satisfying}}\#{\rm{cols}}(M)+\#{\rm{rows}}(M)=k+1\}.$$

The symbols #cols(*M*) and #rows(*M*) denote the number of columns and rows of matrix *M*, respectively. Based on the concept of submatrices, we define the following set of coefficients, which is important in computing ${\omega }^{{\mathbb{X}}\oplus {\mathbb{Z}}}$:

10 $${s}_{k}=\,\max \,\{\Vert M\Vert \,:M\in {\rm{SUB}}(U,k)\},$$

where ||*M*|| is the operator norm equal to the maximum singular value of *M*, and the maximum is optimized over all submatrices of class *k* of *U*. By construction we have ${s}_{1}\le \cdots \le {s}_{d}=1$. In^16^ it is proved that

11 $${\omega }^{{\mathbb{X}}\oplus {\mathbb{Z}}}=\{1\}\oplus s,\,s=({s}_{1},{s}_{2}-{s}_{1},\,\ldots ,\,{s}_{d}-{s}_{d-1},0,\ldots ,0).$$

We append *d* − 1 0 s to *s* to make ${\omega }^{{\mathbb{X}}\oplus {\mathbb{Z}}}$ a 2*d*-dimensional vector. We remark that the vector *s* is not necessarily sorted in descending order, but we can use the “flattening” process described in the first section to make it so. To summarize, the direct-sum majorization uncertainty relation is characterized in the following theorem, originally proved in^16^.

#### **Theorem 1**

*Let* ${\mathbb{X}}$ *and* ${\mathbb{Z}}$ *be two nondegenerate and noncommuting observables on H. For any state* $\rho \in  {\cal{D}} (H)$, it holds that $p({\mathbb{X}}|\rho )\oplus q({\mathbb{Z}}|\rho )\,\prec \,{\omega }^{{\mathbb{X}}\oplus {\mathbb{Z}}}$, where ${\omega }^{{\mathbb{X}}\oplus {\mathbb{Z}}}$ is defined in Eq. (11).

## Entanglement Detection

An entanglement detector decides whether a given bipartite state is separable by providing a condition that is satisfied by all separable states, and if violated, witnesses entanglement. In this section, we design a detection method based on the direct-sum majorization bound described above. As majorization relations, our detector actually provides a set of conditions whose number will grow with the dimension of the state. We first describe a majorization bound for all separable states. Then we show how this bound serves as a detector.

### Majorization bounds

If an observable ${\mathbb{X}}$ is degenerate, the definition of $p({\mathbb{X}}|\rho )$, given in Eq. (5), is not unique, since the spectral decomposition is not unique. By combining eigenstates with the same eigenvalue, however, there exists a unique spectral decomposition of the form ${\mathbb{X}}=\sum _{i}\,{\lambda }_{i}{P}_{i}$, with ${\lambda }_{i}\ne {\lambda }_{{i}^{\text{'}}}$ for $i\ne {i}^{\text{'}}$ and *P*_*i*_ are orthogonal projectors of maximum rank^30^. Under this convention, we define for degenerate observable ${\mathbb{X}}$ the distribution *p*_*i*_ = Tr[*P*_*i*_*ρ*]. Our entanglement detection method relies on the degeneracy properties of the product observables on bipartite systems. It is possible that for two non-commuting observables ${{\mathbb{X}}}_{A}$ and ${{\mathbb{X}}}_{B}$, their product ${{\mathbb{X}}}_{A}\otimes {{\mathbb{X}}}_{B}$ is degenerate. Consequently, it may happen that ${{\mathbb{X}}}_{A}\otimes {{\mathbb{X}}}_{B}$ and ${{\mathbb{Z}}}_{A}\otimes {{\mathbb{Z}}}_{B}$ have a common eigenstate, and this eigenstate is an entangled pure state. In such cases, the probabilities $p({{\mathbb{X}}}_{A}\otimes {{\mathbb{X}}}_{B}|{\rho }_{AB})$ and $p({{\mathbb{Z}}}_{A}\otimes {{\mathbb{Z}}}_{B}|{\rho }_{AB})$ will reflect the stated difference and may be capable of detecting entanglement. As an example, consider the Pauli Z operator *σ*_*z*_ on system *A* and *B*. The product observable on *AB* is given by ${\sigma }_{z}\otimes {\sigma }_{z}$. The spectral decomposition of ${\sigma }_{z}\otimes {\sigma }_{z}$ is (under our convention)

12 $${\sigma }_{z}\otimes {\sigma }_{z}=(|00\rangle \langle 00|+|11\rangle \langle 11|)-(|01\rangle \langle 01|+|10\rangle \langle 10|),$$

Similarly, we have $${\sigma }_{x}\otimes {\sigma }_{x}={P}_{+}-{P}_{-}$$, where $${P}_{+}=|++\rangle \langle ++|+|--\rangle \langle --|$$ and $${P}_{-}=\,|+-\rangle \langle +-|+$$$$|-+\rangle \langle -+|$$, $$|+\rangle =(|0\rangle +|1\rangle /\sqrt{2}$$, $$|-\rangle =(|0\rangle -|1\rangle /\sqrt{2}$$. There exists no state $${\rho }_{A}$$ that can result in certain outcomes for both *σ*_*x*_ and *σ*_*z*_, because they do not commute. But there do exist an entangled state |Ψ〉 that can give certain outcomes for both $${\sigma }_{x}\otimes {\sigma }_{x}$$ and $${\sigma }_{z}\otimes {\sigma }_{z}$$, as they commute. By the Schmidt decomposition, they can be expressed in the same eigenbases which are possibly entangled.

Let ${\mathbb{X}}{}_{A}$ and ${\mathbb{X}}{}_{B}$ be two full rank observables on *A* and *B*, respectively. Assume their spectral decompositions are given by

13 $${{\mathbb{X}}}_{A}=\mathop{\sum }\limits_{i=1}^{d}\,{\alpha }_{i}|{x}_{i}^{A}\rangle \langle {x}_{i}^{A}|,\,{{\mathbb{X}}}_{B}=\mathop{\sum }\limits_{i=1}^{d}\,{\beta }_{i}|{x}_{i}^{B}\rangle \langle {x}_{i}^{B}|.$$

Performing the product measurement ${{\mathbb{X}}}_{A}\otimes {{\mathbb{X}}}_{B}$ on a bipartite state *ρ*_*AB*_, we obtain a joint distribution

14 $$p(i,j)=\langle {x}_{i}^{A}{x}_{j}^{B}|{\rho }_{AB}|{x}_{i}^{A}{x}_{j}^{B}\rangle .$$

As ${{\mathbb{X}}}_{A}\otimes {{\mathbb{X}}}_{B}$ might be degenerate, some elements *p*(*i*, *j*) are grouped together since they belong to the same eigenvalue. We denote by $p({{\mathbb{X}}}_{A}\otimes {{\mathbb{X}}}_{B}|{\rho }_{AB})$ the joint distribution after grouping. If we perform local measurements, we obtain marginal distributions $p({{\mathbb{X}}}_{A}|{\rho }_{A})$ and $p({{\mathbb{X}}}_{B}|{\rho }_{B})$. It is proved in [^30^, Lemma 1] that the joint distribution of a product state is majorized by the distribution of its marginal, which we restate here for completeness.

#### **Lemma 2**

*Let* ${\rho }_{AB}={\rho }_{A}\otimes {\rho }_{B}$ *be a product state and let* ${{\mathbb{X}}}_{A}$ *and* ${{\mathbb{X}}}_{B}$ *be two observables on systems H*_*A*_ *and H*_*B*_, *respectively. Then*

15 $$p({{\mathbb{X}}}_{A}\otimes {{\mathbb{X}}}_{B}|{\rho }_{A}\otimes {\rho }_{B})\,\prec \,p({{\mathbb{X}}}_{A}|{\rho }_{A}),$$

16 $$p({{\mathbb{X}}}_{A}\otimes {{\mathbb{X}}}_{B}|{\rho }_{A}\otimes {\rho }_{B})\,\prec \,p({{\mathbb{X}}}_{B}|{\rho }_{B}).$$

Intuitively, this is because for the product observable ${{\mathbb{X}}}_{A}\otimes {{\mathbb{X}}}_{B}$, its eigenstates are possibly entangled, and thus product state gives uncertain outcomes, however it is possible that the reduced state gives certain outcome for the corresponding local measurements.

Now we consider the effect of several product observables. Let ${{\mathbb{X}}}_{A}$ and ${{\mathbb{Z}}}_{A}$ be two observables on *A*, ${{\mathbb{X}}}_{B}$ and ${{\mathbb{Z}}}_{B}$ be two observables on *B*, respectively. For arbitrary product state ${\rho }_{AB}={\rho }_{A}\otimes {\rho }_{B}$, we obtain from Lemma 2 that

17 $$p({{\mathbb{X}}}_{A}\otimes {{\mathbb{X}}}_{B}|{\rho }_{A}\otimes {\rho }_{B})\,\prec \,p({{\mathbb{X}}}_{A}|{\rho }_{A}),\,p({{\mathbb{Z}}}_{A}\otimes {{\mathbb{Z}}}_{B}|{\rho }_{A}\otimes {\rho }_{B})\,\prec \,p({{\mathbb{Z}}}_{A}|{\rho }_{A}).$$

As the direct-sum operation preserves the majorization order^31^, we have

18 $$p({{\mathbb{X}}}_{A}\otimes {{\mathbb{X}}}_{B}|{\rho }_{A}\otimes {\rho }_{B})\oplus p({{\mathbb{Z}}}_{A}\otimes {{\mathbb{Z}}}_{B}|{\rho }_{A}\otimes {\rho }_{B})\,\prec \,p({{\mathbb{X}}}_{A}|{\rho }_{A})\oplus p({{\mathbb{Z}}}_{A}|{\rho }_{A}).$$

The RHS. of Eq. (18) is the direct-sum of two distributions. By the virtue of Theorem 1, it holds that

19 $$\forall \,{\rho }_{A}\in  {\cal{D}} ({H}_{A}),\,\,p({{\mathbb{X}}}_{A}|{\rho }_{A})\oplus p({{\mathbb{Z}}}_{A}|{\rho }_{A})\,\prec \,{\omega }^{{{\mathbb{X}}}_{A}\oplus {{\mathbb{Z}}}_{A}}.$$

Combining Eqs. (18) and (19), we reach the following statement for arbitrary product states ${\rho }_{A}\otimes {\rho }_{B}$, one has

20 $$p({{\mathbb{X}}}_{A}\otimes {{\mathbb{X}}}_{B}|{\rho }_{A}\otimes {\rho }_{B})\oplus p({{\mathbb{Z}}}_{A}\otimes {{\mathbb{Z}}}_{B}|{\rho }_{A}\otimes {\rho }_{B})\,\prec \,{\omega }^{{{\mathbb{X}}}_{A}\oplus {{\mathbb{Z}}}_{A}}.$$

The majorization relation derived in Eq. (20) holds for product states. Now we show that this relation actually holds for arbitrary separable states. We are actually interested in the optimal state that majorizes all possible probability distributions $p({{\mathbb{X}}}_{A}\otimes {{\mathbb{X}}}_{B}|{\rho }_{AB})\oplus p({{\mathbb{Z}}}_{A}\otimes {{\mathbb{Z}}}_{B}|{\rho }_{AB})$ induced by performing the measurements ${{\mathbb{X}}}_{A}\otimes {{\mathbb{X}}}_{B}$ and ${{\mathbb{Z}}}_{A}\otimes {{\mathbb{Z}}}_{B}$ on *separable* states *ρ*_*AB*_. Such a state can be defined as

21 $${\omega }_{{\rm{SEP}}}^{({{\mathbb{X}}}_{A}{{\mathbb{X}}}_{B})\oplus ({{\mathbb{Z}}}_{A}{{\mathbb{Z}}}_{B})}\,:=\sup \{p({{\mathbb{X}}}_{A}\otimes {{\mathbb{X}}}_{B}|{\rho }_{AB})\oplus p({{\mathbb{Z}}}_{A}\otimes {{\mathbb{Z}}}_{B}|{\rho }_{AB})\,:{\rho }_{AB}\in {\rm{SEP}}({H}_{A}:{H}_{B})\},$$

where SEP(*H*_*A*_: *H*_*B*_) is the set of separable states of the bipartite space ${H}_{A}\otimes {H}_{B}$. Let Ω_SEP_ be the (*d* + 1)-dimensional vector for constructing the supremum ${\omega }_{{\rm{SEP}}}^{({{\mathbb{X}}}_{A}{{\mathbb{X}}}_{B})\oplus ({{\mathbb{Z}}}_{A}{{\mathbb{Z}}}_{B})}$ before the flattening process. That is, ${\omega }_{{\rm{SEP}}}^{({{\mathbb{X}}}_{A}{{\mathbb{X}}}_{B})\oplus ({{\mathbb{Z}}}_{A}{{\mathbb{Z}}}_{B})}$ is obtained from Ω_SEP_ using Eq. (3) after the flattening process. We now show by contradiction that each element of Ω_SEP_ is achieved by some pure product state. Let *μ*_*l*_ be the *l*-th component of Ω_SEP_, where *l* ≤ *d* + 1. By Eq. (2), we can assume without loss of generality that *μ*_*l*_ is achieved by some separable state ${\hat{\rho }}_{AB}=\sum _{k}\,{\lambda }_{k}|{\phi }_{k}^{A}\rangle \langle {\phi }_{k}^{A}|\otimes |{\phi }_{k}^{B}\rangle \langle {\phi }_{k}^{B}|$, where $\{|{\phi }_{k}^{A}\rangle \}$ and $\{|{\phi }_{k}^{B}\rangle \}$ are orthonormal bases of *A* and *B*, respectively. Denote by *I* (*J*) be subsets of distinct index pairs from $\{1,\ldots ,d\}\times \{1,\ldots ,d\}$, and by |*I*| (|*J*|) the size (number of elements) of *I* (*J*). We assume the two probability sequences achieving *μ*_*l*_ are given by *I* and *J* satisfying |*I*| + |*J*| = *l*. That is,

22 $${\mu }_{l}=\sum _{(i,j)\in I}\,p(i,j)+\sum _{(m,n)\in J}\,q(m,n),$$

where *p* and *q* are the joint distributions given by product measurements ${{\mathbb{X}}}_{A}\otimes {{\mathbb{X}}}_{B}$ and ${{\mathbb{Z}}}_{A}\otimes {{\mathbb{Z}}}_{B}$, respectively. From the linearity of the trace function, we have

23 $$p(i,j)=\langle {x}_{i}^{A}{x}_{j}^{B}|{\hat{\rho }}_{AB}|{x}_{i}^{A}{x}_{j}^{B}\rangle =\sum _{k}\,{\lambda }_{k}|\langle {x}_{i}^{A}{x}_{j}^{B}|{\phi }_{k}^{A}{\phi }_{k}^{B}\rangle {|}^{2},$$

24 $$q(m,n)=\langle {z}_{m}^{A}{z}_{n}^{B}|{\hat{\rho }}_{AB}|{z}_{m}^{A}{z}_{n}^{B}\rangle =\sum _{k}\,{\lambda }_{k}|\langle {z}_{m}^{A}{z}_{n}^{B}|{\phi }_{k}^{A}{\phi }_{k}^{B}\rangle {|}^{2}.$$

Thus

25 $${\mu }_{l}=\sum _{(i,j)\in I}\,p(i,j)+\sum _{j\in J}\,q(m,n)=\sum _{k}\,{\lambda }_{k}(\sum _{(i,j)\in I}\,|\langle {x}_{i}^{A}{x}_{j}^{B}|{\phi }_{k}^{A}{\phi }_{k}^{B}\rangle {|}^{2}+\sum _{(m,n)\in J}\,|\langle {z}_{m}^{A}{z}_{n}^{B}|{\phi }_{k}^{A}{\phi }_{k}^{B}\rangle {|}^{2})$$

26 $$\le \mathop{{\rm{\max }}}\limits_{|{\phi }_{k}^{A}{\phi }_{k}^{B}\rangle }\sum _{(i,j)\in I}\,|\langle {x}_{i}^{A}{x}_{j}^{B}|{\phi }_{k}^{A}{\phi }_{k}^{B}\rangle {|}^{2}+\sum _{(m,n)\in J}\,|\langle {z}_{m}^{A}{z}_{n}^{B}|{\phi }_{k}^{A}{\phi }_{k}^{B}\rangle {|}^{2}.$$

That is to say, if ${\hat{\rho }}_{AB}$ achieves *μ*_*l*_, then ${\hat{\rho }}_{AB}$ must be a pure product state, otherwise we can find a pure state which gives larger *μ*_*l*_ by simply choosing the eigenstate of ${\hat{\rho }}_{AB}$ with the largest eigenvalue. To summarize, we reduce the optimization over all separable states required in Eq. (21) to the optimization over all pure product states:

27 $${\omega }_{{\rm{SEP}}}^{({{\mathbb{X}}}_{A}{{\mathbb{X}}}_{B})\oplus ({{\mathbb{Z}}}_{A}{{\mathbb{Z}}}_{B})}=\sup \{p({{\mathbb{X}}}_{A}\otimes {{\mathbb{X}}}_{B}|{\phi }_{AB})\oplus p({{\mathbb{Z}}}_{A}\otimes {{\mathbb{Z}}}_{B}|{\phi }_{AB})\,:\phi =|{\phi }_{A}\rangle \langle {\phi }_{A}|\otimes |{\phi }_{B}\rangle \langle {\phi }_{B}|\}.$$

For an arbitrary separable state (be it pure or not) ${\rho }_{AB}$, it then holds that

28 $$p({{\mathbb{X}}}_{A}\otimes {{\mathbb{X}}}_{B}|{\rho }_{AB})\oplus p({{\mathbb{Z}}}_{A}\otimes {{\mathbb{Z}}}_{B}|{\rho }_{AB})\,\prec \,{\omega }_{{\rm{SEP}}}^{({{\mathbb{X}}}_{A}{{\mathbb{X}}}_{B})\oplus ({{\mathbb{Z}}}_{A}{{\mathbb{Z}}}_{B})}\,\prec \,{\omega }^{{{\mathbb{X}}}_{A}\oplus {{\mathbb{Z}}}_{A}},$$

where the first relation follows from the definition of *ω*_SEP_ , and the second relation follows from Eqs. (27) and (20). We have the following theorem.

#### **Theorem 3**

*Let* ${{\mathbb{X}}}_{A}\otimes {{\mathbb{X}}}_{B}$ *and* ${{\mathbb{Z}}}_{A}\otimes {{\mathbb{Z}}}_{B}$ *be two product observables. For arbitrary separable state* ${\rho }_{AB}\in  {\cal{D}} ({H}_{A}\otimes {H}_{B})$, *it holds that*

29 $$p({{\mathbb{X}}}_{A}\otimes {{\mathbb{X}}}_{B}|{\rho }_{AB})\oplus p({{\mathbb{Z}}}_{A}\otimes {{\mathbb{Z}}}_{B}|{\rho }_{AB})\,\prec \,{\omega }^{{{\mathbb{X}}}_{A}\oplus {{\mathbb{Z}}}_{A}},$$

where ${\omega }^{{{\mathbb{X}}}_{A}\oplus {{\mathbb{Z}}}_{A}}$ is defined in Eq. (11). Similarly, one has

30 $$p({{\mathbb{X}}}_{A}\otimes {{\mathbb{X}}}_{B}|{\rho }_{AB})\oplus p({{\mathbb{Z}}}_{A}\otimes {{\mathbb{Z}}}_{B}|{\rho }_{AB})\,\prec \,{\omega }^{{{\mathbb{X}}}_{B}\oplus {{\mathbb{Z}}}_{B}}.$$

### The detection framework

Theorem 3 states that majorization of $p({{\mathbb{X}}}_{A}\otimes {{\mathbb{X}}}_{B}|{\rho }_{AB})\oplus p({{\mathbb{Z}}}_{A}\otimes {{\mathbb{Z}}}_{B}|{\rho }_{AB})$ by ${\omega }^{{{\mathbb{X}}}_{A}\oplus {{\mathbb{Z}}}_{A}}$ is a necessary condition for the separability of *ρ*_*AB*_ and its violation signals the existence of entanglement. This statement provides an operational method of entanglement detection. Given a bipartite state ${\rho }_{AB}\in  {\cal{D}} ({H}_{A}\otimes {H}_{B})$, we first calculate the direct-sum probability distribution $p({{\mathbb{X}}}_{A}\otimes {{\mathbb{X}}}_{B}|{\rho }_{AB})\oplus p({{\mathbb{Z}}}_{A}\otimes {{\mathbb{Z}}}_{B}|{\rho }_{AB})$ induced by the product measurements ${{\mathbb{X}}}_{A}\otimes {{\mathbb{X}}}_{B}$ and ${{\mathbb{Z}}}_{A}\otimes {{\mathbb{Z}}}_{B}$ by sampling from the source multiple times and gathering statistics. Then we investigate the majorization relation between this quantity and ${\omega }^{{{\mathbb{X}}}_{A}\oplus {{\mathbb{Z}}}_{A}}$. If ${\omega }^{{{\mathbb{X}}}_{A}\oplus {{\mathbb{Z}}}_{A}}$ does not majorize the direct-sum distribution, then we conclude that *ρ*_*AB*_ is entangled. However, if ${\omega }^{{{\mathbb{X}}}_{A}\oplus {{\mathbb{Z}}}_{A}}$ majorizes the distribution, we can say nothing about *ρ*_*AB*_: it can be separable, it can also be entangled.

The proposed method is a collection of detectors. Indeed, Theorem 3 states the following fact. For arbitrary separable state *ρ*_*AB*_ and arbitrary $k\in \{1,\,\ldots ,\,2d\}$, it holds that

31 $$\mathop{\sum }\limits_{i=1}^{k}\,{[p({{\mathbb{X}}}_{A}\otimes {{\mathbb{X}}}_{B}|{\rho }_{AB})\oplus p({{\mathbb{Z}}}_{A}\otimes {{\mathbb{Z}}}_{B}|{\rho }_{AB})]}_{i}^{\downarrow }\le \mathop{\sum }\limits_{i=1}^{k}\,{[{\omega }^{{{\mathbb{X}}}_{B}\oplus {{\mathbb{Z}}}_{B}}]}_{i}^{\downarrow }.$$

As the first and the last *d* inequalities are trivial, we have *d* − 1 effective inequalities in total, thus *d* − 1 detectors. Violation of any of these inequalities is sufficient to detect entanglement in a given state.

### Example

Here we give an example illustrating how to use our proposed method to detect the entanglement of Werner states with Pauli *σ*_*x*_ and *σ*_*z*_ observables. First, using the construction method discussed around Eq. (11), we obtain

32 $${\omega }^{{\sigma }_{x}\oplus {\sigma }_{z}}=(1,1/\sqrt{2},1-1/\sqrt{2},0).$$

The Werner family of two-qubit states^32^ is of the form:

33 $${\rho }^{w}(p)\,:\,=p|\Phi \rangle \langle \Phi |+(1-p)\frac{1}{4},$$

where *p* ∈ [0, 1], $|\Phi \rangle =(|00\rangle +|11\rangle )/\sqrt{2}$ and 1 is the identity operator. It is known that *ρ*^*w*^(*p*) is entangled if and only if *p* > 1/3. If we perform the measurements of the product observables ${\sigma }_{x}\otimes {\sigma }_{x}$ and ${\sigma }_{z}\otimes {\sigma }_{z}$ on a system in state *ρ*^*w*^(*p*), we obtain the same probability distribution by Eq. (12), i.e.,

34 $$p({\sigma }_{x}\otimes {\sigma }_{x}|{\rho }^{w}(p))=p({\sigma }_{z}\otimes {\sigma }_{z}|{\rho }^{w}(p))=(\frac{1+p}{2},\frac{1-p}{2}).$$

As so, the direct-sum of these two probability distributions is

35 $$p({\sigma }_{x}\otimes {\sigma }_{x}|{\rho }^{w}(p))\oplus p({\sigma }_{x}\otimes {\sigma }_{x}|{\rho }^{w}(p))=(\frac{1+p}{2},\frac{1+p}{2},\frac{1-p}{2},\frac{1-p}{2}).$$

According to Theorem 3, *ρ*^*w*^(*p*) violates the separability condition of Eq. (29) if ${\omega }^{{\sigma }_{x}\oplus {\sigma }_{z}}$ fails to majorize the direct-sum quantity in Eq. (35), which occurs when

36 $$\frac{1+p}{2}+\frac{1+p}{2} > 1+\frac{1}{\sqrt{2}},$$

resulting $p > 1/\sqrt{2}$. That is to say, our method can detect the entanglement of *ρ*^*w*^(*p*) for $p > 1/\sqrt{2}$. Obviously, our obtained bound is not tight for the Werner states. However, by optimizing over all possible observables, we can possibly improve the bound for Werner states. Our main point of this example is show that our framework can detect entanglement, say for the Werner states when *p* is large enough. It remains as an interesting problem to figure out for what states our detection framework has good performance.

## Conclusions

In this paper, we have studied the relationship between direct-sum majorization formulation of uncertainty relations and entanglement. We have designed entanglement detection methods based on such a formulation. Our detectors provide a set of sufficient conditions for detecting entanglement whose number grows linearly with the dimension of the state being detected. The proposed entanglement detection methods are of practical importance, as they are experimental friendly and relatively easy to implement.

Several interesting problems remain open. What’s the relation between our entanglement detection method based on direct-sum majorization and that based on tensor-product majorization^28^? Can we obtain stronger detection method by considering a tomographically complete set of observables? We hope the results presented here can stimulate further investigations on the relations among uncertainty relations, majorization, and entanglement.
